# Analysis of the Retinal Microcirculation in Chinese Patients With Vogt–Koyanagi–Harada Disease by Optical Coherence Tomography Angiography

**DOI:** 10.1167/tvst.14.11.22

**Published:** 2025-11-17

**Authors:** Fanfan Huang, Chong Tang, Shiyao Tan, Rong Hu, Jingjie Hu, Peizeng Yang

**Affiliations:** 1 Ophthalmology Medical Center, The First Affiliated Hospital of Chongqing Medical University, Chongqing Key Laboratory for the Prevention and Treatment of Major Blinding Eye Diseases, Chongqing Branch (Municipality Division) of National Clinical Research Centre for Ocular Diseases, Chongqing, China; 2 Department of Ophthalmology, West China Hospital, Sichuan University, Chengdu, Sichuan, China; 3 Department of Optometry and Visual Science, West China Hospital, Sichuan University, Chengdu, Sichuan, China; 4 Department of Ophthalmology, Daping Hospital, Army Medical University, Chongqing, China; 5 Department of Ophthalmology, Joint Research Laboratory for Ocular Immunology and Retinal Injury Repair, The First Affiliated Hospital of Zhengzhou University, Henan International, Henan Province Eye Hospital, Zhengzhou, China

**Keywords:** optical coherence tomography angiography, perfusion, retinal vessel density, vogt–koyanagi–harada disease

## Abstract

**Purpose:**

The dynamic changes of retinal microcirculation in Chinese Vogt–Koyanagi–Harada (VKH) patients have not yet been described. This study aimed to explore changes in retinal microcirculation among Chinese with VKH disease using optical coherence tomography angiography (OCTA) technology to determine whether patient outcomes improve due to therapeutic interventions.

**Methods:**

The study included 43 VKH patients and 43 normal controls. Vessel density (VD), perfusion density (PD), and the foveal avascular zone (FAZ) area, perimeter, and circularity were measured by OCTA. Clinical and demographic characteristics of VKH patients and visual acuity changes were recorded. All patients were treated with prednisone in combination with cyclosporine.

**Results:**

Before treatment, VD of the parafoveal and full area, PD of the parafoveal area, and visual acuity were significantly decreased in VKH patients compared to controls. These parameters increased significantly to normal values after 2 months of treatment and maintained a normal level thereafter. VD of the foveal area, PD of the foveal and full area, and FAZ area, perimeter, and circularity did not differ from those of normal eyes and showed no significant changes following treatment. A negative correlation was found between visual acuity and PD of the parafoveal and full area and FAZ area and circularity.

**Conclusions:**

Decreased retinal VD and PD were observed in Chinese VKH patients, which returned to normal values following treatment. OCTA analysis may be a useful method to follow the response of VKH patients to treatment.

**Translational Relevance:**

OCTA can objectively evaluate the retinal microcirculation and is an effective technology to track the response of VKH patients to treatment.

## Introduction

Vogt–Koyanagi–Harada (VKH) disease is a systemic autoimmune disorder that typically manifests as a bilateral granulomatous panuveitis and can affect skin, the auditory–vestibular system, and meninges.^1^ The ocular inflammation primarily causes choroiditis with subsequent involvement of the retina and the anterior segment.^2^ In the early stage of VKH disease, ocular findings such as serous retinal detachment (SRD) and swelling of the optic disc head are characteristic features.^3^ Middle-aged adults are most likely to be affected, and the disease is more frequently seen in Asians than in Caucasians.^4^

Optical coherence tomography angiography (OCTA) has been developed to observe changes in the vascular structure of the retina and choroid.^5^^,^^6^ This non-invasive examination can be repeated frequently, unlike other angiographic tests. OCTA has been used to assess parameters such as the foveal avascular zone (FAZ).^7^ Vessel density (VD) and perfusion density (PD) of the superficial capillary plexus (SCP) can also be quantified with this technique and may reveal microvascular involvement of the retina in certain eye diseases.^8^ Although early studies have described the use of OCTA in the choroid of VKH patients,^9^^,^^10^ few reports have addressed parameters such as VD, PD, and FAZ of the SCP in VKH^11^^,^^12^; therefore, this was the subject of the study described here. In this prospective interventional longitudinal study, we measured VD, PD, and FAZ before, during, and after treatment during a period of approximately 18 months.

## Methods

The present study adhered to the tenets of the Declaration of Helsinki and included VKH patients that were referred to the Ophthalmology Department of the First Affiliated Hospital of Chongqing Medical University (Chongqing, China) from January 2021 to February 2023. All patients signed an informed consent for this study, which was approved by the Institutional Review Board of this university. All patients were in the acute uveitic phase at baseline, characterized by bilateral granulomatous panuveitis with choroiditis and SRD in most cases. Staging followed the established criteria of the Diagnostic Criteria for VKH Disease (DCV)^13^ and the Revised Diagnostic Criteria for VKH Disease (RDC).^14^ Patients were subsequently treated according to our published standard treatment regimen^15^ as detailed below and were followed for a mean duration of 8.4 months (range, 6–20). Patients with media opacities or other structural abnormalities that could have affected the OCTA analysis, such as cataract, corneal disease, retinal, and vitreous hemorrhage, were excluded. Patients with glaucomatous neuropathy, diabetic retinopathy, and hypertensive retinopathy that might have caused microvascular damage to the retina were also excluded. After applying the above inclusion and exclusion criteria, 43 patients with VKH disease were eligible for the study. Best-corrected visual acuity (BCVA), intraocular pressure (IOP), slit-lamp biomicroscopic examination and ophthalmoscopy were performed by our routine examiners,^16^ and Snellen acuity was converted to the logarithm of minimal angle of resolution (logMAR). The fundus was evaluated by color fundus photography (IMAGEnet R4 Lite; Topcon, Tokyo, Japan), fundus fluorescence angiography (FFA; SPECTRALIS retinal angiography; Heidelberg Engineering, Heidelberg, Germany), indocyanine green angiography (ICGA; Heidelberg Engineering), and B-scan ultrasonography (SW-2100; Suoer, Tianjin, China), all scored by two operators. The OCTA images taken before treatment were considered as the baseline, and all patients underwent at least three OCTA examinations during overall follow-up. This study recruited controls as a convenience sample of healthy volunteers. The age- and gender-matched control group (*n* = 43) showed no ocular abnormalities and was examined under the same circumstances as we used for the VKH patients.

Our standard regimen for the treatment of VKH disease includes an initial dose of systemic prednisone of 0.5 to 0.8 mg/kg/d in combination with cyclosporine.^17^ After 1 to 2 weeks, the prednisone was tapered to a maintenance dose of 15 to 20 mg/d over 4 to 6 months. This dose was used for 4 to 6 months and then gradually tapered to a stop over the following 4 to 6 months. The initial dosage of cyclosporine (2–4 mg/kg/d) was used and gradually tapered if the initial dosage was more than 2 mg/kg/d. Posterior sub-Tenon's injection of triamcinolone acetonide (20 mg) was used for patients with severe SRD. Topical corticosteroids and mydriatic and cycloplegic agents were used to treat patients with anterior segment inflammation.

**Figure 1.**
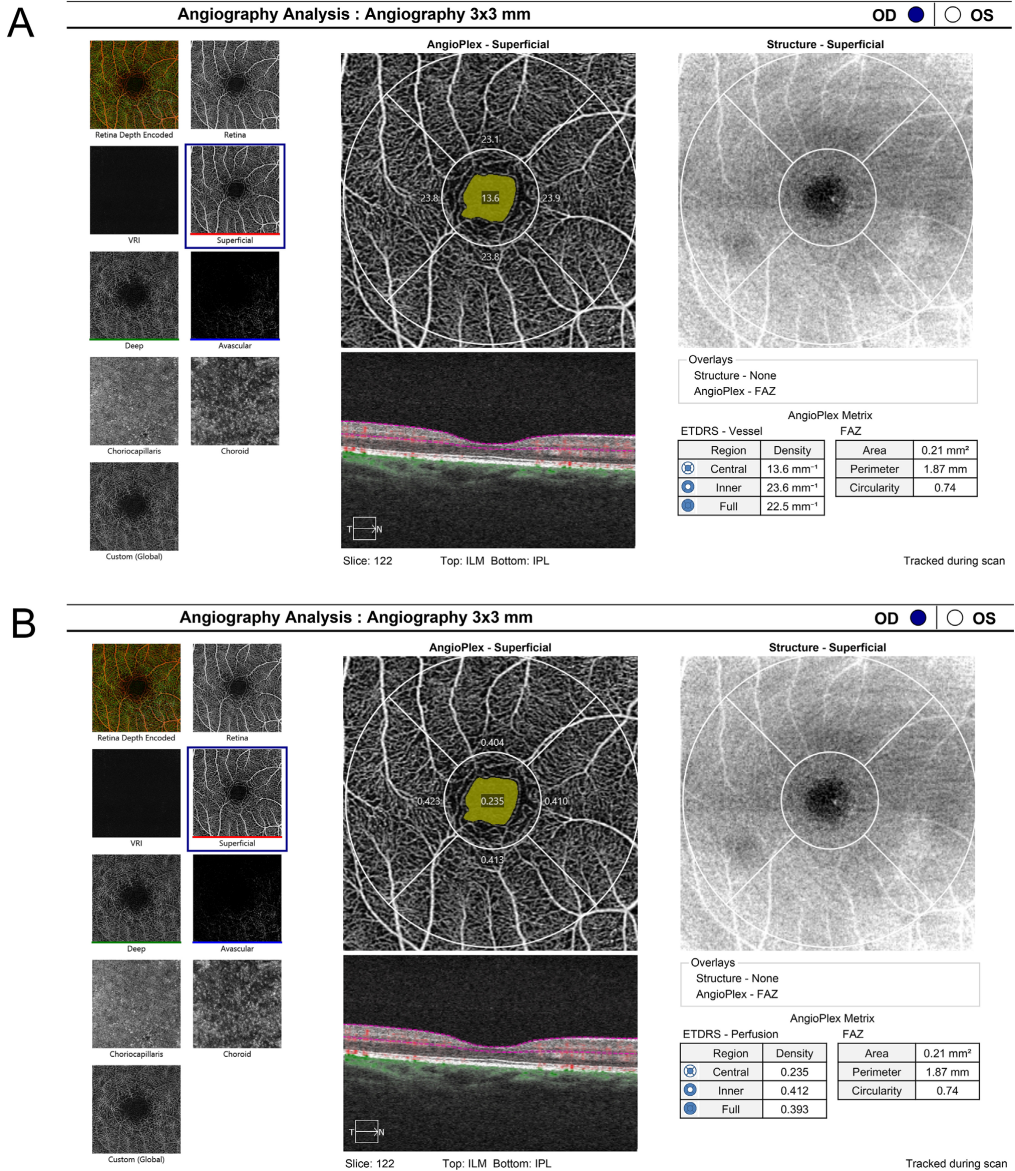
The grid of VD and PD of SCPs. Shown are the outer circle with a diameter of 3 mm (full area), an area between the 1-mm-diameter and 3-mm-diameter circles (parafoveal area), an inner circle with a diameter of 1 mm (foveal area). (**A**) Average VD was automatically calculated for each sector. (**B**) Average PD was automatically calculated for each sector, and the *yellow area* represents the FAZ area.

Patients were regularly examined using OCTA before treatment (*n* = 43 eyes) and at 2 months (±2 weeks; *n* = 40 eyes), 6 months (±4 weeks; *n* = 38 eyes), 12 months (±6 weeks; *n* = 30 eyes), and 18 months (±8 weeks; *n* = 24 eyes) after treatment. The measurements were taken from 8 AM to 12 AM to avoid diurnal variations in axial length and IOP,^18^ and the OCTA was performed by two experienced technicians independently who were not aware of the diagnosis of patients; they used the CIRRUS HD-OCT 5000 with AngioPlex 9.5 software (Carl Zeiss Meditec, Dublin, CA). Dilation of pupils is not necessary for OCTA examinations, which significantly encourages patient compliance. All of the OCTA images were acquired simultaneously, based on 245 × 245 A-scans of horizontal lines of 3 × 3 mm centered on the foveal area while all 6 × 6 mm images were excluded.^19^ The SCP was measured from the internal limiting membrane to the inner plexiform layer. VD was quantified as the total length of perfused vessels identified within the 3 × 3-mm foveal map, expressed in mm/mm^2^ (mm⁻^1^). PD was defined as the proportion of the assessed area showing blood circulation, expressed as a percentage.^20^ VD emphasizes vascular structure, whereas PD highlights functional flow. The foveal map was divided into total 3 × 3-mm area (full area), area between the 1-mm-diameter and 3-mm-diameter circles (parafoveal area) and the central 1 × 1-mm area (foveal area). This division is the same as used in the Early Treatment Diabetic Retinopathy Study (ETDRS) grading of diabetic retinopathy with inner and outer circles (shown in Fig. 1).^21^ We also collected the FAZ area (mm^2^), perimeter (mm), and circularity of each patient. Data from normal controls were obtained under the same circumstances. During OCTA testing, the ZEISS OCT incorporates FastTrac retinal tracking technology to minimize the influence of eye movement artifacts and to decrease rescanning times; images with serious layered errors and signal strength of 5 or less were excluded.^22^ To address potential artifacts from SRD, segmentation boundaries were reviewed for accuracy. Minor adjustments were restricted to FAZ boundaries only when fluid caused misalignment, but extensive manual segmentation was avoided to maintain reproducibility.

For statistical analysis we used the right eye from each patient. Statistical analyses were performed using SPSS Statistics 23.0 (IBM, Chicago, IL) and SAS 9.4 (SAS Institute, Cary, NC). Graphs were drawn using Prism 6.02 (GraphPad Software, Boston, MA). Categorical variables of basic information are shown as numbers and percentages, whereas the continuous variables VD, PD, and FAZ are represented by mean value ± SD. Independent-samples *t*-tests were used to analyze the difference between patients and controls, and generalized linear mixed modeling revealed changes in patient OCTA results longitudinally. A *P* value was accepted as statistically significant when it was less than 0.05.

## Results

### Demographics and Clinical Manifestations

Forty-three right eyes from 43 patients (18 men, 25 women; mean age, 41.3 ± 12.7 years) with VKH disease were enrolled in this study and were followed-up with a mean duration of 8.4 months (range, 6–20). Right eyes from 43 age- and gender- matched (*P* = 0.938 and *P* = 0.828, respectively) normal individuals (19 men, 24 women; mean age, 40.5 ± 9.6 years) were included as controls. The IOP values were all within the normal range. For statistical analysis, we used the right eye from each patient.

The initial and subsequent clinical manifestations were assessed for the whole group of 43 VKH patients (Table 1). Twenty eyes (46.5%) showed the presence of anterior chamber cells, and 17 eyes (39.5%) showed keratic precipitates at the same time. All eyes with VKH disease had posterior segment involvement such as diffuse choroiditis (100%). FFA showed subsequent pooling of the dye in the subretinal space in 25 eyes (58.1%) and optic disk hyperfluorescence in 18 eyes (41.9%). ICGA showed hypofluorescent spots in 43 eyes (100%). Ultrasonography showed diffuse choroidal thickening in 38 eyes (88.4%) and SRD in 39 eyes (90.7%). The four eyes without SRD met DCV and RDC via choroiditis, ICGA findings, and extraocular signs. During follow-up, 10 eyes (23.3%) developed sunset glow fundus and one eye (2.3%), a typical refractory case, showed Koeppe nodules and choroidal neovascularization. In the week before disease onset, 12 patients (27.9%) felt fatigue frequently, five patients (11.6%) consumed alcohol, and five patients (11.6%) had recently experienced an influenza infection. Two patients did not show any general manifestations, but headache, nausea, tinnitus, hearing loss, alopecia, poliosis, nuchal rigidity, and vitiligo were observed, respectively, in 76.7%, 9.3%, 51.2%, 27.9%, 23.3%, 14.0%, 30.2%, and 4.7% of the other VKH disease patients. Four patients (9.3%) showed a disease recurrence.

**Table 1.** Clinical Manifestation of the 43 VKH Patients

	Initial Manifestation, *n* (%)	Manifestation During Follow-Up, *n* (%)
Anterior segment		
Anterior cells	20 (47)	8 (19)
Keratic precipitates	17 (40)	4 (9)
Koeppe nodules	0	1 (2)
Posterior segment		
Choroiditis	43 (100)	3 (7)
Retinal detachment	39 (91)	4 (9)
Subretinal pooling (FFA)	25 (58)	2 (5)
Optic disk hyperfluorescence (FFA)	18 (42)	0
Hypofluorescent spots (ICGA)	43 (100)	4 (9)
Choroidal thickening (B-scan)	38 (88)	2 (5)
Sunset glow fundus	0	10 (23)
Choroidal neovascularization	0	1 (2)
Before disease onset		
Fatigue	12 (28)	—
Drinking alcohol	5 (12)	—
Influenza	5 (12)	—
Extraocular manifestation		
Headache	18 (42)	33 (77)
Nausea	2 (5)	4 (9)
Tinnitus	21 (49)	22 (51)
Hearing loss	4 (9)	12 (28)
Alopecia	6 (14)	10 (23)
Poliosis	2 (5)	6 (14)
Nuchal rigidity	10 (23)	13 (30)
Vitiligo	0	2 (5)

FFA, fundus fluorescence angiography; ICGA, indocyanine green angiography.

### Changes in VD, PD, and FAZ

**Figure 2.**
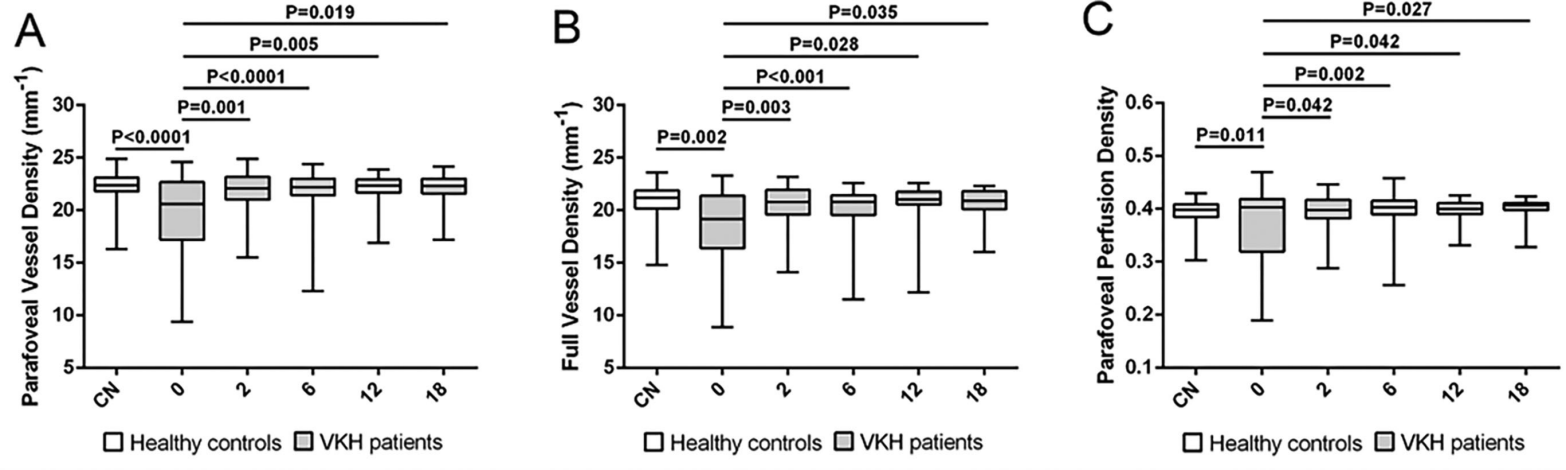
The dynamic changes in VD and PD in VKH patients. (**A**) VD of the parafoveal area of VKH patients and comparisons with the healthy controls. (**B**) VD of the full area in VKH patients and comparisons with the healthy controls. (**C**) PD of the parafoveal area in VKH patients and comparisons with the healthy controls.

**Figure 3.**
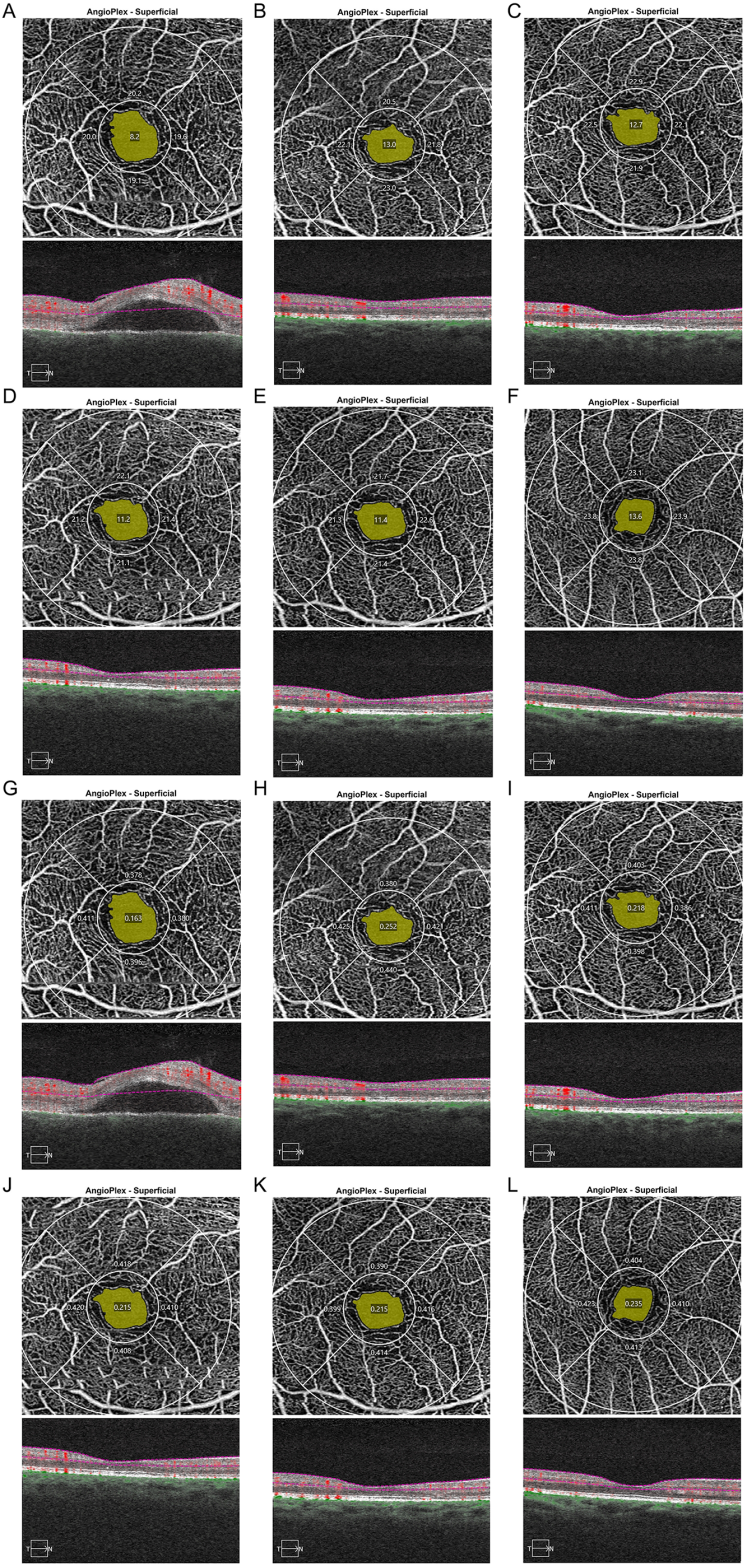
VD and PD changes in a 49-year-old male VKH patient during follow-up. (**A**) VD before treatment. (**B**) VD after 2 months of treatment. (**C**) VD after 6 months of treatment. (**D**) VD after 11 months of treatment. (**E**) VD after 17 months of treatment. (**F**) VD of a healthy control. (**G**) PD before treatment. (**H**) PD after 2 months of treatment. (**I**) PD after 6 months of treatment. (J) PD after 11 months of treatment. (**K**) PD after 17 months of treatment. (**L**) PD of a healthy control.

Medical history and OCTA data were collected from VKH patients at first presentation and during follow-up visits at 2 months, 6 months, 12 months, and 18 months. We started with 43 eyes and were still evaluating 24 eyes from 24 patients at the 18-month time point (Table 2). In the cohort of eyes with VKH disease at the first visit and prior to initiating treatment, VD of the parafoveal and full area (*P* < 0.001 and *P* = 0.002, respectively), together with PD of the parafoveal area (*P* = 0.011), showed significantly lower values compared to healthy controls. The VD of the parafoveal and full area and PD of the parafoveal area increased markedly at 2 months following treatment (*P* = 0.001, *P* = 0.003, and *P* = 0.042, respectively) and maintained a normal level thereafter (shown in Fig. 2). No significant differences were observed in VD of the foveal area, PD of the foveal and full area, or the FAZ area, perimeter, and circularity (all P > 0.05) between the VKH patients and normal controls following treatment. A typical example of normal control and dynamic changes of VD and PD in a 49-year-old male VKH patient at different time points is shown in the Figure 3.

**Table 2.** OCTA Results in VKH Patients and Normal Controls

		Mean ± SD				
		Time Post-Treatment (Months)				
	Normal Controls (*n* = 43 Eyes)	0 (*n* = 43 Eyes)	2 (*n* = 40 Eyes)	6 (*n* = 38 Eyes)	12 (*n* = 30 Eyes)	18 (*n* = 24 Eyes)
VD						
Foveal (mm^−1^)	10.06 ± 3.59	10.14 ± 4.19	9.94 ± 2.49	9.28 ± 2.42	11.08 ± 3.15	9.39 ± 2.71
Parafoveal (mm^−1^)	22.04 ± 1.74	19.57 ± 3.95^a^	21.80 ± 2.03^b^	21.68 ± 2.45^b^	22.16 ± 1.35^b^	22.18 ± 1.36^c^
Full (mm^−1^)	20.69 ± 1.81	18.60 ± 3.79^a^	20.48 ± 2.01^b^	20.11 ± 2.34^b^	20.61 ± 2.08^c^	20.75 ± 1.33^c^
PD						
Foveal	0.17 ± 0.06	0.19 ± 0.08	0.18 ± 0.04	0.17 ± 0.04	0.19 ± 0.06	0.17 ± 0.05
Parafoveal	0.39 ± 0.03	0.37 ± 0.07^d^	0.39 ± 0.03^c^	0.40 ± 0.04^b^	0.40 ± 0.02^c^	0.40 ± 0.02^c^
Full	0.37 ± 0.03	0.36 ± 0.07	0.37 ± 0.03	0.37 ± 0.04	0.38 ± 0.02	0.37 ± 0.02
FAZ						
Area (mm^2^)	0.32 ± 0.12	0.30 ± 0.09	0.31 ± 0.09	0.34 ± 0.08	0.30 ± 0.10	0.34 ± 0.10
Perimeter (mm)	2.40 ± 0.50	2.69 ± 2.20	2.40 ± 0.37	2.52 ± 0.35	2.30 ± 0.57	2.52 ± 0.41
Circularity	0.67 ± 0.10	0.65 ± 0.09	0.66 ± 0.10	0.66 ± 0.10	0.65 ± 0.09	0.67 ± 0.07

a *P* < 0.01 (comparison between patients and normal controls using the independent-samples *t*-test).

b *P* < 0.01 (comparison between VKH patients at first presentation and their visits at 2 months, 6 months, 12 months, and 18 months using the generalized linear mixed model).

c *P* < 0.05 (comparison between VKH patients at first presentation and their visits at 2 months, 6 months, 12 months, and 18 months using the generalized linear mixed model).

d *P* < 0.05 (comparison between patients and normal controls using the independent-samples *t*-test).

### Visual Outcome

**Figure 4.**
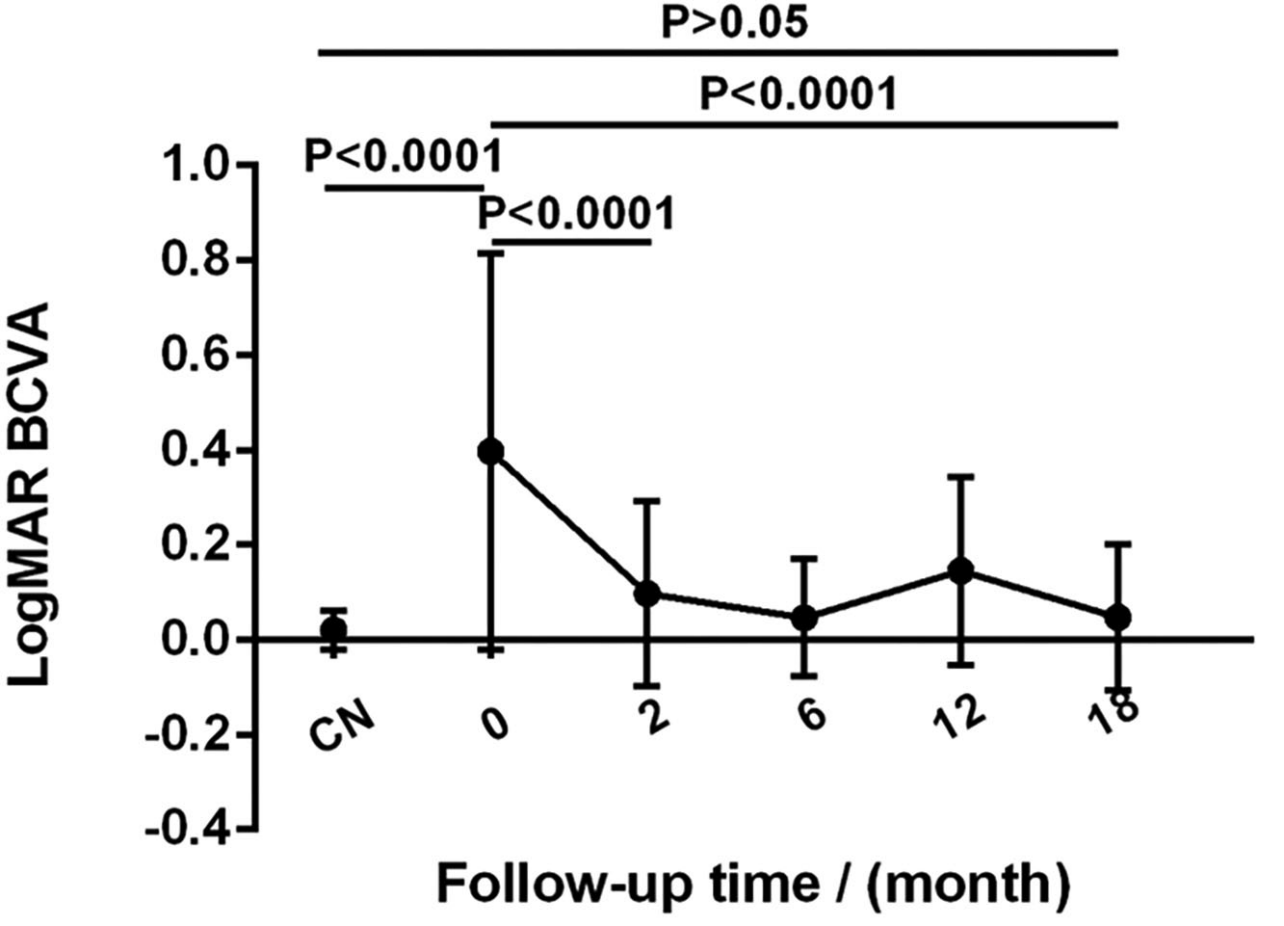
The changes in BCVA of healthy controls (CN) and VKH patients during follow-up.

During the 1.5-year follow-up, most patients showed an improvement of their BCVA. Before treatment, 23 eyes (53.5%) had a logMAR value greater than 0.3 (20/40), showing that patients’ visual acuity was obviously poorer than that of the normal eyes (*P* < 0.001). At 2 months, the average logMAR value of VKH patients improved from 0.41 (20/50; range, 20/20 to 20/2000) to 0.11 (20/25; range, 20/16 to 20/200; *P* < 0.001) (shown in Fig. 4). At the last follow-up, the average logMAR value had significantly improved to 0.05 (20/23; range, 20/20 to 20/100; *P* < 0.001) and showed no difference compared with healthy control eyes (*P* > 0.05). Spearman correlation analysis showed that PD of the parafoveal and full area, FAZ area, and FAZ circularity were negatively associated with the logMAR values of patients, whereas the other OCTA parameters showed no significant correlation with BCVA (Table 3).

**Table 3.** Spearman Correlation Analysis of BCVA and OCTA Parameters

	Vessel Density			Perfusion Density			FAZ		
	Foveal	Parafoveal	Full	Foveal	Parafoveal	Full	Foveal	Parafoveal	Full
Coefficient	−0.122	−0.248	−0.251	−0.218	−0.476^a^	−0.423^a^	−0.315^a^	−0.004	−0.398^a^

a *P* < 0.05.

## Discussion

This study used OCTA to show the dynamic changes in the retinal microcirculation of Chinese VKH patients. In the SCP, decreased retinal VD and PD were observed in VKH patients, which returned to normal values following 2 months of treatment with systemic corticosteroids and cyclosporine. No changes could be detected in VD and PD of the foveal area, PD of the full area, or the FAZ area, perimeter, and circularity. We found that visual acuity was associated with PD of the parafoveal and full area, FAZ area, and FAZ circularity. Apart from this, we investigated longitudinal changes of the ocular and systemic manifestations in VKH patients by using routine and auxiliary tests and found that patients generally suffered from choroiditis (100%). Headache (76.7%) was the most common symptom mentioned by the patients. Most of the VKH patients recovered, and their BCVA showed a significant improvement at the last visit.

Based on the comparison among popular OCTA algorithms, the Zeiss CIRRUS HD-OCT 5000 has good repeatability and showed the highest VD values of the retinal SCP with a moderate image quality.^23^^,^^24^ In our study, a marked decrease was found in retinal VD of the parafoveal, full area, and PD of the parafoveal area before treatment, showing that, although the choroid is the primary target of autoimmune attack, VKH disease also has a circulatory disturbance in the retina, as well.^11^^,^^25^ At different stages of VKH, the retinal SCP may be influenced by SRD and subretinal fluid, which can increase the metabolic transmission distance from choroid to the retina, thereby leading to retinal hypoxia and abnormal retinal revascularization.^26^ SRD may affect registration, but patterns persisted in non-SRD eyes, suggesting intrinsic microvascular involvement. Moreover, the retina is part of the central nervous system and has a similar high metabolism as the brain. When the circulation of the choroid and the deep retinal capillary plexus (DCP) is impaired, supply does not equal demand of the retinal SCP, and this can result in excitotoxic cell death^27^ and a decrease in vessel and perfusion density. In previous studies, retinal metabolism was shown to be abnormal in the acute uveitic stage but returned to a normal level in VKH patients who recovered without permanent structural damage,^28^ which is consistent with our findings using OCTA. Different from a domestic study that used average values to represent the OCTA data in the acute and quiescent stages,^12^ our parameters measured at each follow-up point could clearly reflect the dynamic changes in a patient's condition. However, unlike a recent study on OCTA analysis of inactive VKH patients,^11^ we did not find a significantly decreased SCP VD and PD after treatment. A possible explanation may be the fact that all of our patients were treated within 2 months after the onset of VKH disease, and our study also included 10 VKH patients with permanent anatomical damage, such as “sunset glow fundus,” who had irreversible damage to their retinal oxygen metabolism. Hypoxia is associated with the formation of neovessels, and relief from a microcirculation disturbance may lead to retinal arteriolar dilation and constriction.^29^ SCP VD and PD might show no differences after the neutralization of these autoregulatory responses. VD and PD of the foveal area seem less important, as the central fovea is mainly avascular. PD of the full area and the FAZ area, perimeter, and circularity showed no differences between patients before and after treatment, and did not differ from normal eyes. Reasons for these results might be the strict exclusion of eyes and the relatively small sample size. Previous studies emphasized that the DCP might be a sensitive indicator of inflammatory status due to its proximity to the choroid and the vulnerable location, which could cause ischemia.^25^^,^^30^ In our study, we focused on the superficial retinal layer because it is influenced less by the superposition artifacts and the outer retinal layers when compared to the DCP. A 2024 meta-analysis synthesized OCTA finding across VKH stages and devices, reporting depressed macular perfusion in active disease, thicker CT and SFCT in the active phase, and a modest increase in FAZ during remission versus controls while acknowledging substantial between-study heterogeneity, particularly for FAZ and choriocapillaris indices. We interpret the apparent discrepancies as consequences of device and threshold diversity and variable artifact handling in pooled studies, whereas the shared signal, worse macular perfusion with activity, and improvement with quiescence are consistent. Future work should harmonize OCTA metric definitions and prioritize DCP-focused longitudinal analyses to refine biomarker performance in VKH.^31^ Visual outcomes were correlated with retinal SCP and PD of the parafoveal and full area, together with FAZ area and circularity, but not with VD. An enlargement of the FAZ area can be observed in some ocular diseases with chorioretinal atrophy but was not obvious in the VKH patients.^32^ The FAZ area may be affected by several factors and has been shown to be quite variable among healthy individuals.^33^

We would like to point out some limitations in our study. First, our study was on a voluntary basis and may thus include a certain selection bias. We had a relatively small sample size of ideal VKH patients due to the use of strict inclusion criteria such as the onset time of VKH, follow-up duration, and general condition. Second, patients are referred to our department from all over China, and, due to logistic and financial reasons, some patients could not have an OCTA examination at each programmed time point. Third, although two professional testers performed the OCTA examinations separately and manual FAZ adjustments enhanced accuracy, it may have introduced minor interoperator variability. Serous detachments may introduce segmentation artifacts, and future studies could incorporate advanced algorithms for correction. Apart from these limitations we do believe that SCP values obtained by OCTA are useful parameters in following the response of VKH patients to treatment.

## Conclusions

In summary, this investigation revealed that Chinese patients with VKH disease exhibited a significant reduction in retinal vessel and perfusion densities, which returned to normal levels after a 2-month treatment regimen with prednisone and cyclosporine. Importantly, OCTA has proven invaluable in quantitatively monitoring these improvements, affirming its critical role in evaluating the effectiveness of therapeutic interventions in VKH. These findings not only highlight the potential of OCTA as a diagnostic tool but also suggest a positive correlation between treatment and the restoration of normal retinal microcirculatory function in VKH patients.
